# Symptom burden in chronic kidney disease; a population based cross sectional study

**DOI:** 10.1186/s12882-017-0638-y

**Published:** 2017-07-10

**Authors:** Sameera Senanayake, Nalika Gunawardena, Paba Palihawadana, Palitha Bandara, Rashan Haniffa, R Karunarathna, Priyantha Kumara

**Affiliations:** 1 Family Health Bureau, Colombo, Sri Lanka; 2 World Health Organization, Colombo, Sri Lanka; 3 Epidemiology Unit, Colombo, Sri Lanka; 4 North Central Provincial Directors Office, Anuradhapura, Sri Lanka; 5 Centre for Tropical Medicine, Nuffield Department of Medicine, University of Oxford, Oxford, UK

**Keywords:** Chronic kidney disease, Symptom burden, Sri Lanka, Chronic kidney disease of unknown aetiology

## Abstract

**Background:**

Physical and psychological symptoms are among main manifestations of Chronic Kidney Disease (CKD). This study aimed to assess the symptom burden and self-perceived severity of symptoms among CKD patients living in a district in Sri Lanka.

**Method:**

A community based cross-sectional study included a sample of randomly selected 1174 CKD patients from all 19 Medical Officer of Health areas in the district of Anuradhapura. Trained para-medical staff visited the households and administered the locally validated questionnaire to assess the presence and severity of symptoms. The inquiry was on 25 symptoms in a 5 point Likert scale indicating the severity during the previous week. Symptom burden score was constructed by summing each symptom severity score which ranged from 0 to 125.

**Results:**

A total of 1118 CKD patients participated with a response rate of 95.2%. The mean age was 58.3 (SD 10.8) years and 62.7% were males. A majority were in CKD stage 4 (58.3%). Bone/joint pain was the most experienced symptom (87.6%; 95%CI 85.6–89.5). Loss of libido was the most severe symptom. The median symptom burden score was 35.0 (IQR 20.0–50.0). Multiple linear regression revealed education up to Advanced Level (β −9.176), CKD stage V (β 3.373), being dialyzed (β 20.944), comorbidities (β 4.241) and being employed (β −9.176) to be significant predictors of symptom burden.

**Conclusions:**

Patients in all stages of CKD experience high symptom burden warranting rigorous measures to relieve symptoms and to improve the well-being of CKD patients.

**Electronic supplementary material:**

The online version of this article (doi:10.1186/s12882-017-0638-y) contains supplementary material, which is available to authorized users.

## Background

Over the years, chronic kidney disease (CKD) has emerged as a major public health problem world over, with adverse physical, psychological and economic outcomes. World Health Report (2002) and Global Burden of Disease (GBD) project state that diseases of the kidney and urinary tract contribute to the global burden of diseases, with approximately 850,000 deaths every year and 15,010,167 disability-adjusted life years (DALYs) globally [1].

Chronic Kidney Disease can be caused by various etiological agents and is often a consequence of diabetes and hypertension. CKD which cannot be attributed to any known aetiology is termed Chronic Kidney Disease of uncertain aetiology (CKDu) [2]. Records show an exponential increase in the number of cases of CKDu in Sri Lanka since the early 1990’s [3]. Of the 26 districts of Sri Lanka, the Anuradhapura district carries the highest CKD burden and is currently experiencing an epidemic of CKDu [4]^.^

Over the last few decades symptom management and end-of-life strategies have been focused mainly on cancer patients and little attention has been paid to patients with other life threatening conditions [5, 6]. However, studies show that patients with other life threatening conditions such as CKD also experience a similar degree of symptom distress as cancer patients [7]. The symptom burden of a disease plays a central role in the patient’s experience of the disease and troublesome physical and psychological symptoms are among the main manifestations of CKD.

Common symptoms experienced by CKD patients are fatigue, pruritus, irritability, anxiety and nausea. An assessment of the symptom burden of all CKD patients is very important in clinical management. However, evidence shows that healthcare providers frequently under-recognize and under-treat the physical symptoms, with patients subsequently experiencing immense physical and physiological trauma [8]. Additionally, although it is generally considered that the early stages of CKD are asymptomatic, recent evidence suggests that the symptom burden has no relationship to the stage of CKD [9].

In this milieu, the aim of this study was to assess the prevalence and symptom burden of CKD patients in all stages of the disease. This study offers new insight into the symptom burden in different stages of the disease, which severely affects the quality of life of CKD patients. These results could be utilized for planning and development of patient-centered services for CKD patients.

## Methods

### Patient selection

A population based descriptive cross-sectional study was conducted in the district of Anuradhapura in the North Central Province (NCP) of Sri Lanka. The study population consisted of confirmed CKD patients who were over 18 years old, with documented evidence of CKD, living in the Anuradhapura district. The data collectors assessed the eligibility of patients by reviewing their clinical records. Informed consent was obtained from those eligible for participation in the study.

The sample size (n) for estimating the proportion of the population with CKD was calculated using the formula $n={z}^2\widehat{p}\left(1-\widehat{p}\right)/{M}^2$. The standard normal deviate (z) was 1.96 for a confidence interval of 95%, the expected proportion of CKD patients with symptoms $\left(\widehat{p}\right)$ was estimated at 50% and the margin of error (*M*) was 3%. A final sample size of 1174 was obtained after a 10% non-response rate was factored in.

The study was conducted in all nineteen Medical Officer of Health (MOH) areas of the Anuradhapura district. The number of participants to be included from each MOH area was based on probability proportionate to the size of CKD patients registered in each of the MOH areas. The required number of participants from each MOH area was selected using the simple random sampling method. The population based CKD register – which records the patients with a confirmed diagnosis of CKD from renal clinics in hospitals of the NCP since 2003 – was used as the sampling frame. The register was obtained from the office of the Provincial Director of Health Services.

### Assessment of symptom prevalence, severity and burden

Symptom prevalence, severity and burden were assessed using the locally developed and validated CKD Symptom Index – Sri Lanka (CKDSI-Sri Lanka) (Additional file 1). The instrument was confirmed as having a good construct validity and test re-test reliability [10].

The CKDSI –Sri Lanka consists of a checklist of 25 symptoms. Each one requires a response of ‘No’ if the patient did not experience the particular symptom during the 7 days prior to the time of inquiry and the response ‘Yes’ if they did experience it during that time period. If the response is positive the patient is then asked to rate the severity of the symptom on a 5 point Likert scale.

Prevalence of the symptoms of CKD was estimated based on the number of study participants who responded that they experienced a particular symptom in the timeframe specified by the CKDSI-Sri Lanka. The prevalence of the symptoms was estimated with the corresponding 95% confidence interval. In assessing the symptom burden, the severity rate of each symptom, 1 to 5, was treated as a score. Those who did not experience the symptom were given a score of zero. The symptom burden score for each respondent was the sum of the symptom severity score for each of the symptoms included in the CKDSI-Sri Lanka. The possible score ranged from zero to 125.

### Survey administration and data collection

Trained Public Health Inspectors visited the households and administered the locally validated questionnaire to assess the presence and severity of symptoms. A two day training session was conducted for the data collectors by the PI which included a mock data collection training session in the Madirigiriya MOH area of the Polonnaruwa district. All possible measures were taken to ensure the quality of information gathered while collecting data. Initially, the eligibility of the selected study participants was assessed by the data collectors and if found eligible they were informed the purpose of the study and were invited to participate in the study.

After patient enrollment, the data collector collected basic demographic data from the patient and administered the CKDSI-Sri Lanka. Clinical, biochemical and treatment related information was extracted from the patient’s personal medical record. The latest available serum creatinine value within 3 months of data collection was used to calculate the eGFR of the patient. Modification of Diet in Renal Disease (MDRD) equation was used for this purpose.

### Statistical analysis

Statistical analysis was done by using SPSS version 20.0. The symptom burden score of the study population was non-normally distributed indicating a skewed distribution. Thus non-parametric tests were used in the bivariate analysis (Mann-Whitney U test and Spearman’s r correlation). A *p* value of less than 0.05 was considered statistically significant. The following variables were analyzed with the symptom burden of CKD; age, education level, income status, employment status, the presence of comorbidities, the number of years since diagnosing CKD and CKD stage. In order to explore how each socio demographic, as well as disease related characteristics, influence the symptom burden when the effects of other factors are controlled, multiple linear regression analysis was performed. Independent variables used for these analyses were the variables that derived a probability value of less than 0.2 in the bivariate analysis.

## Results

### Patient characteristics

Out of the 1174 participants selected to be included in the study, 56 (4.8%) did not participate in the study giving a response rate of 95.2%. The mean age of the study population was 58.4 years (SD 10.8). There was a preponderance of males among the study population (62.7%, *N* = 701). The mean eGFR of the population was 31.8 (SD 20.2) ml/min/1.73 m2. The mean number of years since being diagnosed with CKD was 4.1 (SD 3.2) years. The majority of participants were in the later stages, stage 4 or beyond, of CKD (*n* = 820; 73.3%). All 38 (3.4%) of the stage 5 participants who were undergoing dialysis, were on haemo-dialysis. Chronic Kidney Disease of Unknown origin (CKDu) was the cause of the CKD in most of the study population (*n* = 489; 43.7%). The second most common cause was hypertension (*n* = 360; *n* = 32.2%).

### Prevalence of symptoms

The most prevalent symptoms among the study population during the 1 week under inquiry were bone/joint pain (87.6%; 95% CI 85.6–89.5), feeling irritable (78.6%; 95% CI 76.2–81.0), muscle cramps (77.5%; 95% CI 75.0–79.9), lack of energy (75.7%; 95% CI 73.2–78.2) and difficulty in sleeping (68.5%; 95% CI 65.8–71.2). The least prevalent symptoms were diarrhea (5.8%; 95% CI 4.4–7.2), vomiting (13.7%; 95% CI 11.7–15.7), hiccups (16.5%; 95% CI 14.3–18.6) and change in skin color (17.5%; 95% CI 15.3–19.8) (Table 1).

**Table 1:** : Prevalence of Symptoms of CKD among the Study Population During the Period of 1 Week

Symptom	(*N* = 1118)	Prevalence	95% Confidence Interval	
			Lower %	Upper %
Loss of appetite	718	64.2	61.4	67.0
Nausea	386	34.5	31.7	37.3
Vomiting	153	13.7	11.7	15.7
Diarrhea	65	5.8	4.4	7.2
Lethargy	641	57.3	54.4	60.2
Changes in skin color	196	17.5	15.3	19.8
Swelling of arms or legs	596	53.3	50.4	56.2
Difficulty in breathing	644	57.6	54.7	60.5
Hiccups	184	16.5	14.3	18.6
Difficulty keeping legs still	259	23.2	20.7	25.7
Numbness/tingling of hands and feet	353	31.5	28.8	34.2
Lack of energy	846	75.7	73.2	78.2
Trouble with memory	678	60.6	57.8	63.5
Weight loss	375	33.5	30.8	36.3
Bone/joint pain	979	87.6	85.6	89.5
Muscle cramps	866	77.5	75.0	79.9
Difficulty concentrating	670	59.9	57.1	62.8
Dry skin	518	46.3	43.4	49.3
Itching	517	46.2	43.3	49.2
Feeling sad	687	61.4	58.6	64.3
Difficulty sleeping	766	68.5	65.8	71.2
Feeling irritable	879	78.6	76.2	81.0
Loss/ decreased libido	494	44.2	41.3	47.1
Impotence	488	43.6	40.7	46.6
Heartburn	620	55.5	52.5	58.4

Prevalence of symptoms of the study population by different stages of CKD is summarized in Table 2. CKD stages were categorized as “Early stage”, “Stage 4”, “Stage 5 – Non Dialysis” and “Stage 5 – Dialysis”. “Early stage” included stages 1, 2 and 3. The symptoms that showed a high prevalence in the week under inquiry among the study population in early stages of CKD were, bone/joint pain (85.7%; 95% CI 81.4–90.0), feeling irritable (77.2%; 95% CI 72.1–82.3) and muscle cramps (71.4%; 95% CI 65.9–76.9). Difficulty keeping legs still (100.0%; 95% CI 100.0–100.0), bone/joint pain (97.4%; 95% CI 93.4–100.0) and feeling irritable (94.7%; 95% CI 88.8–100.0) were the highest prevalent symptoms among those in stage 5 and on dialysis.

**Table 2:** : Prevalence of Symptoms of CKD among the Study Population during the Period of One Week by their Stage in the CKD Disease

Symptom	Early stage (*N* = 259)		Stage 4 (*N* = 629)		Stage 5 – Non dialysis (*N* = 153)		Stage 5 – Dialysis (*N* = 38)	
	n (%)	95% CI	n (%)	95% CI	n (%)	95% CI	n (%)	95% CI
Loss of appetite	150 (57.9)	51.9–63.9	410 (65.2)	61.5–68.9	104 (68.0)	60.6–75.4	32 (84.2)	72.6–95.8
Nausea	77 (29.7)	24.1–35.3	206 (32.8)	29.1–36.5	67 (43.7)	35.8–51.6	22 (57.9)	42.2–73.6
Vomiting	25 (9.6)	6.0–13.2	77 (12.2)	9.6–14.8	38 (24.8)	18.0–31.6	10 (26.3)	12.3–40.3
Diarrhea	10 (3.8)	1.5–6.1	35 (5.5)	3.7–7.3	13 (8.5)	4.1–12.9	06 (15.8)	4.2–27.4
Lethargy	133 (51.4)	45.3–57.5	360 (57.2)	53.3–61.1	95 (62.1)	54.4–69.8	32 (84.2)	72.6–95.8
Changes in skin color	29 (11.2)	7.4–15.0	89 (14.1)	11.4–16.8	48 (31.4)	24.0–38.8	22 (57.9)	42.2–73.6
Swelling of arms or legs	104 (40.2)	34.2–46.2	321 (51.0)	47.1–54.9	115 (75.2)	68.4–82.0	21 (81.6)	69.3–93.9
Difficulty in breathing	146 (44.8)	38.7–50.9	338 (55.3)	51.4–59.2	100 (78.4)	71.9–84.9	32 (84.2)	72.6–95.8
Hiccups	29 (11.2)	7.4–15.0	105 (16.7)	13.8–19.6	31 (20.3)	13.9–26.7	13 (34.2)	19.1–49.3
Difficulty keeping legs still	15 (5.8)	3.0–8.6	80 (12.7)	10.1–15.3	126 (82.3)	76.3–88.3	38 (100.0)	100.0–100
Numbness/tingling of hands and feet	46 (17.7)	13.1–22.3	173 (27.5)	24.2–31.2	105 (68.6)	61.2–76.0	29 (76.3)	31.4–63.2
Lack of energy	178 (68.7)	63.1–74.3	467 (74.2)	70.8–77.6	138 (90.2)	85.5–94.9	35 (92.1)	83.5–100.0
Trouble with memory	150 (57.9)	51.9–63.9	369 (58.7)	54.9–62.5	106 (69.3)	62.9–75.7	27 (71.1)	54.7–87.5
Weight loss	84 (32.4)	26.7–38.1	203 (32.3)	28.6–36.0	49 (32.0)	24.2–39.8	23 (60.5)	42.4–78.6
Bone/joint pain	222 (85.7)	81.4–90.0	550 (87.4)	84.8–90.0	139 (90.8)	82.9–98.7	37 (97.4)	93.4–100.0
Muscle cramps	185 (71.4)	65.9–76.9	493 (78.4)	75.2–81.6	121 (79.1)	71.3–86.9	35 (92.1)	85.3–98.9
Difficulty concentrating	146 (56.4)	50.4–62.4	379 (60.3)	56.5–64.1	90 (58.8)	51.1–66.5	31 (81.6)	69.5–93.7
Dry skin	100 (38.6)	32.7–44.5	289 (45.9)	42.0–49.8	87 (55.6)	48.5–62.7	23 (60.5)	41.9–79.1
Itching	106 (40.9)	34.9–46.9	280 (44.5)	40.6–48.4	88 (57.5)	51.3–63.7	22 (57.9)	37.8–78.0
Feeling sad	161 (62.2)	56.3–68.1	374 (59.5)	55.7–63.3	93 (60.8)	52.9–68.7	30 (78.9)	67.3–90.5
Difficulty sleeping	167 (64.5)	58.7–70.3	431 (68.5)	64.9–72.1	110 (71.9)	64.0–79.8	33 (86.8)	77.6–96.0
Feeling irritable	200 (77.2)	72.1–82.3	486 (77.3)	74.0–80.6	124 (81.0)	73.3–88.7	36 (94.7)	88.8–100.0
Loss/ decreased libido	113 (43.6)	37.6–49.6	272 (43.2)	39.3–47.1	70 (45.8)	37.9–53.7	24 (63.2)	47.9–78.5
Impotence	109 (42.1)	36.1–48.1	270 (42.9)	39.0–46.8	70 (45.8)	37.9–53.7	24 (63.2)	47.9–78.5
Heartburn	139 (53.7)	47.6–59.8	342 (54.4)	50.5–58.3	93 (60.8)	53.1–68.5	25 (65.8)	50.7–80.9

*CKD stage was not available in 39 study units

Muscle cramps were perceived as “Severe” by 250 (28.9%) of those who experienced the symptom, while 264 (30.0%) of those who experienced irritability perceived it as “Severe”. Among those who had loss/ decreased libido and impotence, 54.5 and 50.4% perceived it as ‘Very severe’ (Table 3).

**Table 3:** : Perceived Severity of the Symptoms related to CKD

Symptom	n	Perceived severity of the symptoms									
		Very mild		Mild		Moderate		Severe		Very severe	
		**n**	**%**	**n**	**%**	**n**	**%**	**n**	**%**	**n**	**%**
Loss of appetite	718	45	(6.3)	144	(20.1)	328	(45.7)	151	(21.0)	50	(7.0)
Nausea	386	21	(5.4)	124	(32.1)	180	(46.6)	47	(12.2)	14	(3.6)
Vomiting	153	14	(9.2)	58	(37.9)	58	(37.9)	20	(13.1)	03	(2.0)
Diarrhea	65	03	(4.6)	21	(32.3)	26	(40.0)	08	(12.3)	07	(10.8)
Lethargy	641	21	(3.3)	172	(26.8)	279	(43.5)	143	(22.3)	26	(4.1)
Changes in skin color	196	35	(17.9)	107	(54.6)	46	(23.5)	05	(2.6)	03	(1.5)
Swelling of arms or legs	596	55	(9.2)	177	(29.7)	189	(31.7)	137	(23.0)	38	(6.4)
Difficulty in breathing	644	49	(7.6)	272	(42.2)	218	(33.9)	86	(13.4)	19	(3.0)
Hiccups	184	18	(9.8)	85	(46.2)	71	(38.6)	07	(3.8)	03	(1.6)
Difficulty keeping legs still	259	18	(6.9)	104	(40.2)	72	(27.8)	35	(13.5)	30	(11.6)
Numbness/tingling of hands and feet	353	24	(6.8)	87	(24.6)	113	(32.0)	75	(21.2)	54	(15.3)
Lack of energy	846	17	(2.0)	260	(30.7)	347	(41.0)	178	(21.0)	44	(5.2)
Trouble with memory	678	39	(5.8)	183	(27.0)	317	(46.8)	112	(16.5)	27	(4.0)
Weight loss	375	36	(9.6)	169	(45.1)	141	(37.6)	19	(5.1)	10	(2.7)
Bone/joint pain	979	19	(1.9)	220	(22.5)	510	(52.1)	183	(18.7)	47	(4.8)
Muscle cramps	866	46	(5.3)	237	(27.4)	304	(35.1)	250	(28.9)	29	(3.3)
Difficulty concentrating	670	46	(6.9)	241	(36.0)	301	(44.9)	72	(10.7)	10	(1.5)
Dry skin	518	60	(11.6)	208	(40.2)	181	(34.9)	45	(8.7)	24	(4.6)
Itching	517	35	(6.8)	150	(29.0)	184	(35.6)	109	(21.1)	39	(7.5)
Feeling sad	687	49	(7.1)	215	(31.3)	270	(39.3)	134	(19.5)	19	(2.8)
Difficulty sleeping	766	53	(6.9)	168	(21.9)	296	(38.6)	200	(26.1)	49	(6.4)
Feeling irritable	879	63	(7.2)	159	(18.1)	367	(41.8)	264	(30.0)	26	(3.0)
Loss/ decreased libido	494	08	(1.6)	44	(8.9)	76	(15.4)	97	(19.6)	269	(54.5)
Impotence	488	08	(1.6)	53	(10.9)	82	(16.8)	99	(20.3)	246	(50.4)
Heartburn	620	24	(3.9)	198	(31.9)	282	(45.5)	97	(15.6)	19	(3.1)

### Burden of symptoms

The median symptom burden score of CKD was 35.0 (IQR 20.0–50.0) while the mean score was 35.8 (SD 20.0).

In order to explore how each socio demographic, as well as disease related characteristics, influenced the symptom burden of CKD when the effects of other factors are controlled, multiple linear regression analysis was performed. A stepwise procedure was adapted in analysis. The residuals were normally distributed and there was no evidence of heteroscedasticity. Evidence of outliers was assessed by Cook’s distance score and a score less than one was considered appropriate. Multicollinearity was assessed using Variance Inflation Factor (VIF), which should be less than four. The Cook’s distance was 0.014 and the VIF score was less than two.

Table 4 demonstrates significant independent predictors of symptom burden of CKD.

**Table 4:** : Independent Predictors for Symptom Burden of CKD

Parameter	Unstandardized Coefficients		Standardized Coefficients	95% CI		Sig.
	B coefficient	SE	B	Lower	Upper	
(Constant)	32.339	1.680		29.043	35.635	*p* < 0.001
Being Employed	−9.176	1.310	−0.218	−11.747	−6.606	*p* < 0.001
GCE A/L passed^*^	−6.813	3.193	−0.059	−13.078	−0.547	*p* = 0.033
CKD stage 5	3.373	1.610	0.058	0.214	6.533	*p* = 0.036
Being Dialyzed	20.944	3.068	0.190	14.924	26.964	*p* < 0.001
Presence of comorbidities	4.241	1.236	0.096	1.815	6.666	*p* = 0.001

*General Certificate Examination Advanced Level

## Discussion

A review of literature globally reveals considerable variation in the reporting of symptom prevalence across studies. This variation could be partially attributed to the inconsistency seen among the study instruments used. These instruments vary in the number of symptoms included; the types of symptoms assessed - with some assessing either mental or physical symptoms and not both; the time period over which the symptoms were experienced, varying from 1 to 2 days to 1 week; and also in the difference in assessment scales used. These differences in reporting make a comparison between studies difficult and limit the understanding of the symptom burden in the different stages of CKD.

Comparable findings to the current study were evident in a review of literature published by Almutary et al., [11] where nineteen articles had been reviewed from 2006 to 2012. According to the review, fifteen symptoms (out of 30 symptoms) had been experienced by more than 40% of the patients reviewed in the analysis. Similar estimates of high prevalence were found for muscle cramps, lack of energy and difficulty in sleeping [11]. The review had been conducted in studies that had assessed CKD patients in the advanced stages (stage 4 and 5), and since 77% of the patients in the current study were also in the advanced stages of the disease, it is reasonable to assume that the figures are comparable. However, bone/joint pain, which had the highest prevalence in the present study, was found to be less prevalent among the study participants included in the review.

In the present study, the prevalence of symptoms was similar and high, irrespective of the Stage of the CKD. Bone/joint pain (85.7%; 95% CI 81.4–90.0), feeling irritable (77.2%; 95% CI 72.1–82.3) and muscle cramps (71.4%; 95% CI 65.9–76.9) were high and common in the early stages of CKD. Although the limited sample size (*n* = 46) limits valid comparison, Herrera et al., (2014), who conducted a study among Salvadoran farming communities, revealed arthralgia, decreased libido and cramps to be the most prevalent symptoms in the early stages of CKD (stage II and III) [12]. In another study done by Pagels et al., (2015), leg cramps, dry skin, stiff/sore joints and impaired sexual desire/ability were the most prevalent symptoms among 35 CKD patients who were in stages II and III of the disease [13].

Difficulty in keeping the legs still (*n* = 38; 100.0%), bone/ joint pain (*n* = 37; 97.4%) and feeling irritable (*n* = 36; 94.7%) were the highest prevalent symptoms in stage V. This finding is of very important clinical significance. It is generally assumed that patients with End Stage Renal Disease (ESRD) would be subjected to dialysis and will experience a substantial improvement in physical and psychological wellbeing. However the fact that even the CKD patients in Stage V dialysis were experiencing a high prevalence of symptoms in the present study may indicate dialysis inadequacy among the participants.

Based on patient perceptions, the most severe symptom, was loss/ decreased libido followed by impotence. A similar finding has been found in a study done by Rosas and colleagues, where impotence had been reported by 82% of the patients and of them 45% described the symptom to be severe [14].

Alhough one can consider a median score of 35 out of 125 for the symptom burden not to be a ‘major concern’, when it is viewed alongside the fact that the present study included only the CKD patients registered by the health authorities, the symptom burden can be considered unacceptable. The fact that a great majority of the patients had accessed care but were none the less experiencing high prevalence of symptoms may be an indication of inadequate attention by the service providers to the relieving of the patients’ symptom burden. In most developed countries, organized systems to provide pharmacological and non-pharmacological remedies specifically designed to relieve CKD patients of their symptoms have been instituted.

Furthermore, most of the evidence regarding symptomatology in the literature were among CKD patients of known etiology. Contrastingly the etiology in the majority of the patients in the current study was unknown (CKDu); a systematic assessment of symptomatology among CKDu patients has not been carried out elsewhere. It could be that the symptom burden is significantly more among CKDu patients and this warrants further evaluation in the future.

Evidence suggests that the socio-economic culture of a population can affect the symptoms experienced by CKD patients [11]. The low socio-economic conditions in the Anuradhapura district could be favouring a high symptom burden. In addition, since CKD is highly prevalent in the Anuradhapura district, it can be assumed that either a relative, friend or a neighbor of the patient has previously been affected by the disease. When a person is labelled with CKD it is believed that they develop cognitive models about their symptomatology based on their knowledge or from personal experience of others such as family members who are having CKD. It has been found that these cognitive models directly influence the individual’s response to the illness with regard to physical and psychological symptoms [15, 16]. This also could be a reason for the high symptom burden among current study population.

Multivariate analysis revealed that being a CKD stage 5 patient, being dialyzed and presence of comorbidities were significant predictors of having high symptom burden scores while being employed and higher educational status were significant predictors of less symptom burden scores.

Uremia and complications associated with advanced stages of CKD (stage 5 and stage 5 with dialysis) such as anemia, metabolic derangements and fluid retention are the most possible explanation for a high symptom burden in advanced stages of the disease. The finding of low education status to be an independent predictor of high symptom burden while being employed to be an independent predictor of low symptom burden among the current study population was supported by Winkleby et al., [17] and Adler and Ostrove [1] reporting an association between low education status and being unemployed with poor health outcomes [17, 18].

## Conclusion

In conclusion, high symptom burden among CKD patients is a major public health problem in the country. Unacceptable burden of symptoms among CKD patients in all stages of the disease should be brought to the notice of the healthcare providers caring for the CKD patients and health policymakers. It is recommended that guidelines be developed on symptom alleviating therapies and that the renal care providers be trained on the implementing of the therapies. Provisions should be made to specifically serve the groups identified to be vulnerable for high symptom burden.

## Additional File

Additional file 1: Chronic Kidney Disease Symptom Index – Sri Lanka. The symptom index used to assess the prevalence, severity and burden of symptoms among CKD patients in the current study. (PDF 313 kb)

## Abbreviations

CKD: Chronic Kidny Disease
CKDSI-Sri Lanka: Chronic Kidny Disease Symptom Index – Sri Lanka
CKDu: Chronic Kidny Disease of unknown eatiology
DALYs: Disability-adjusted life years
GBD: Global Burden of Disease
MDRD: Modification of Diet in Renal Disease
MOH: Medical Officer of Health
NCP: North Central Province
VIF: Variance Inflation Factor
