# Supplementary material for: Symptom burden in chronic kidney disease; a population based cross sectional study
Source: BMC Nephrol. 2017 Jul 10;18:228. doi: 10.1186/s12882-017-0638-y (PMC5504715; doi:10.1186/s12882-017-0638-y)
Supplement: Additional file 1: — Chronic Kidney Disease Symptom Index – Sri Lanka. The symptom index used to assess the prevalence, severity and burden of symptoms among CKD patients in the current study. (PDF 313 kb) [file 12882_2017_638_MOESM1_ESM.pdf]

Chronic Kidney Disease Symptom Index – Sri Lanka

Below is a list of problems that people with chronic kidney failure may have. For each symptom, please indicate if they had the problem during the past week by circling "yes" or "no." If "yes", ask them to indicate how sever that problem was by circling the appropriate number.

Read to the respondent.
It has been shown that people with chronic kidney disease have various problems related to the disease. All patients will not have all the problems and the perception of the severity of those problems varies with the individual. Now I would like to ask about the problems you have. Please indicate if you experienced each of the following problem during the past week. For all the problems you have, I want you to rate the severity of each in a scale of "Very mild", "Mild", "Moderate", "Severe" and "Very Severe"

During the past week: *Did you experience this problem?*			***If "yes": How severe was the problem?***				
			**Very mild**	**Mild**	**Mode rate**	**Severe**	**Very severe**
1	Loss of appetite	No					
		Yes →	1	2	3	4	5
2	Nausea	No					
		Yes →	1	2	3	4	5
3	Vomiting	No					
		Yes →	1	2	3	4	5
4	Diarrhea	No					
		Yes →	1	2	3	4	5
5	Lethargy	No					
		Yes →	1	2	3	4	5
6	Changes in skin color	No					
		Yes →	1	2	3	4	5
7	Swelling of arms or legs	No					
		Yes →	1	2	3	4	5
8	Difficulty in breathing	No					
		Yes →	1	2	3	4	5
9	Hiccups	No					
		Yes →	1	2	3	4	5
10	Difficulty keeping legs still	No					
		Yes →	1	2	3	4	5
11	Numbness/tingling of hands and feet	No					
		Yes →	1	2	3	4	5
12	Lack of energy	No					
		Yes →	1	2	3	4	5
13	Trouble with memory	No					
		Yes →	1	2	3	4	5
14	Weight loss	No					
		Yes →	1	2	3	4	5
15	Bone/joint pain	No					
		Yes →	1	2	3	4	5
16	Muscle cramps	No					
		Yes →	1	2	3	4	5

17	Difficulty concentrating	No					
		Yes →	1	2	3	4	5
18	Dry skin	No					
		Yes →	1	2	3	4	5
19	Itching	No					
		Yes →	1	2	3	4	5
20	Feeling sad	No					
		Yes →	1	2	3	4	5
21	Difficulty sleeping	No					
		Yes →	1	2	3	4	5
22	Feeling irritable	No					
		Yes →	1	2	3	4	5
23	Loss/ decreased libido	No					
		Yes →	1	2	3	4	5
24	Impotence	No					
		Yes →	1	2	3	4	5
25	Heartburn	No					
		Yes →	1	2	3	4	5
